# Biosorption of Methylene Blue by De-Oiled Algal Biomass: Equilibrium, Kinetics and Artificial Neural Network Modelling

**DOI:** 10.1371/journal.pone.0109545

**Published:** 2014-10-13

**Authors:** Rahulkumar Maurya, Tonmoy Ghosh, Chetan Paliwal, Anupama Shrivastav, Kaumeel Chokshi, Imran Pancha, Arup Ghosh, Sandhya Mishra

**Affiliations:** 1 Discipline of Salt & Marine Chemicals, CSIR - Central Salt & Marine Chemicals Research Institute, G B Marg, Bhavnagar, Gujarat, India; 2 Academy of Scientific & Innovative Research (AcSIR), CSIR - Central Salt & Marine Chemicals Research Institute, G B Marg, Bhavnagar, Gujarat, India; 3 Discipline of Wasteland Research, CSIR - Central Salt & Marine Chemicals Research Institute, G B Marg, Bhavnagar, Gujarat, India; 4 Bioenergy Engineering Research Laboratory, Chemical and Biomolecular Engineering Department, KAIST, Yuseong-gu, Daejeon, South Korea; Glasgow University, United Kingdom

## Abstract

The main objective of the present study is to effectively utilize the de-oiled algal biomass (DAB) to minimize the waste streams from algal biofuel by using it as an adsorbent. Methylene blue (MB) was used as a sorbate for evaluating the potential of DAB as a biosorbent. The DAB was characterized by SEM, FTIR, pH_PZC_, particle size, pore volume and pore diameter to understand the biosorption mechanism. The equilibrium studies were carried out by variation in different parameters, i.e., pH (2–9), temperature (293.16–323.16 K), biosorbent dosage (1–10 g L^−1^), contact time (0–1,440 min), agitation speed (0–150 rpm) and dye concentration (25–2,500 mg L^−1^). MB removal was greater than 90% in both acidic and basic pH. The optimum result of MB removal was found at 5–7 g L^−1^ DAB concentration. DAB removes 86% dye in 5 minutes under static conditions and nearly 100% in 24 hours when agitated at 150 rpm. The highest adsorption capacity was found 139.11 mg g^−1^ at 2,000 mg L^−1^ initial MB concentration. The process attained equilibrium in 24 hours. It is an endothermic process whose spontaneity increases with temperature. MB biosorption by DAB follows pseudo-second order kinetics. Artificial neural network (ANN) model also validates the experimental dye removal efficiency (R^2^ = 0.97) corresponding with theoretically predicted values. Sensitivity analysis suggests that temperature and agitation speed affect the process most with 23.62% and 21.08% influence on MB biosorption, respectively. Dye adsorption capacity of DAB in fixed bed column was 107.57 mg g^−1^ in preliminary study while it went up to 139.11 mg g^−1^ in batch studies. The probable mechanism for biosorption in this study is chemisorptions via surface active charges in the initial phase followed by physical sorption by occupying pores of DAB.

## Introduction

Treatment of wastewater streams has always been an important and challenging area of research and several physico-chemical and biological processes exist for colour removal from various effluents. Most of the industrial effluents i.e. of textile, rubber, paper and printing, cosmetics, food, leather, pharmaceutical, plastics etc. contain large amount of toxic dyes which are mutagenic and carcinogenic to life forms [1].

Common methods for colour removal are coagulation and flocculation [2], biological oxidation and chemical precipitation [3] and activated carbon adsorption [4]; the latter is the preferred method for removing, recovering and recycling of dyes from wastewaters due to its simplicity. However, it is not without its limitations due to the costs involved as well as difficulties in regeneration of the substrate [5], [6]. Therefore, recent interest has shifted to low-cost materials that range from waste products of other industries to naturally abundant biomass such as peanut hull [7], rice husk [2], water hyacinth roots [8], hexane-extracted spent bleaching earth [9], raw and activated date pits [10], guava seeds [11], macroalgae *Sargassum muticum* [12], *Parthenium* plants [13], bacteria and fungi [14] among many others.

Algae are one of the most promising sources of biomass, biofuel, organic fertilizer, protein supplements and high value nutraceutical products due to their benefits like, ability to utilize fresh, marine or wastewater, non-requirement of fertile land, do not compete with food crops and reduce green house gas from the environment [15], [16]. Recent research has been focusing on practicality and sustainability of algal biofuels [16], [17]. Algal biodiesel production generates a large amount of waste de-oiled algal biomass (DAB), which is still the subject of numerous studies including its use as a substrate for bioethanol, biogas in addition to be used as animal/poultry/fish feed, fertilizer, and remediation of dyes & heavy metals [18], [15].

Methylene blue (MB) has been used in the present study to model the dye adsorption capacity of the de-oiled algal biomass. Main objective of the present study is to evaluate the effects of different physicochemical parameters like pH, agitation speed, temperature, dye-biosorbent contact time, adsorbent concentration and initial dye concentration with the performance as predicted through different kinetic models including pseudo-first and second order kinetics and intra particle diffusion. We have also analysed the whole data set using artificial neural network (ANN) to validate the experimental as well as predicted values generated which were found to agree very well with each other.

## Materials and Methodology

### DAB preparation and characterization

The naturally occurring floating algal biomass dominated by *Microspora* sp. (ATCC PTA-12197) was collected from the coastal lagoons of Gujarat (site coordinates 20° 42.391′ N and 70° 54.959′ E) and was first de-oiled through Soxhlet extraction using hexane as the solvent [19]. The algal biomass collected was not an endangered/protected species. No specific permission was required from any authority for collection of said biomass. DAB was oven dried for 48 hours at 80°C to remove any residual hexane and ground for further characterization and experiments. Surface topology of the DAB was observed using a scanning electron microscope (LEO 1430VP, Zeiss, Germany), whereas BET (Brunauer-Emmett-Teller) surface area and total pore volume was obtained using Micromeritics ASAP 2010 V5.02, USA. The particle size distribution was calculated based on differential pore volume of Barrett-Joyner-Halenda (BJH) adsorption and desorption. FT-IR spectrum of DAB was obtained using the KBr disc method (Spectrum GX, Perkin Elmer, USA) with a resolution of 4 cm^−1^ in the range of 400–4000 cm^−1^ region for the examination of surface open functional groups. The point of zero charge (pH_PZC_) was determined by salt addition method [20].

### Adsorption experiments

The effects of pH (2–9), temperature (293.16–323.16 K), shaking (0–150 rpm), initial dye concentration (25–2500 mg L^−1^), contact time (5–1440 min) and biosorbent dosage (1–10 g L^−1^) for the MB removal were investigated. The respective conditions have been mentioned in the relevant figures.

The samples were withdrawn from each flask at 5 minutes interval till an hour and every hour upto 5 hours and the final reading was taken at 24 hours. The samples were centrifuged and MB concentration in the supernatant was determined at 665 nm using UV-Visible Spectrophotometer (Varian Cary-50 Bio, Varian Inc., USA). Except column experiment, all the experiments were carried out in duplicate with a working volume of 200 mL in 500 mL Erlenmeyer flasks at room temperature (27±2°C) and under static conditions unless specified otherwise.

### Adsorption thermodynamics

The changes in enthalpy (ΔH°), entropy (ΔS°), Gibbs free energy (ΔG°) as well as the equilibrium constant (K_c_) for MB biosorption over DAB was calculated using van’t Hoff equation (Equation 3).

The change in free energy is related to the equilibrium constant by the following relationship:
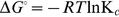
(1)where R is the gas constant (8.314 J mol^−1^ K^−1^) and T is the absolute temperature in K. According to the Gibbs’ free energy equation:



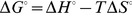
(2)Combining equation 1 and 2, we get:
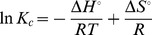
(3)


A plot between ln K_c_ and 1/T gives a linear graph whose intercept and slope yields ΔS°/R and ΔH°/R, respectively.

### Parameters of Kinetic studies

The MB adsorption q_t_ (mg g^−1^) at time t, was calculated using the following equation:
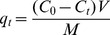
(4)where q_t_ is the dye uptake (mg g^−1^), C_0_ (mg L^−1^) is the initial dye concentration and C_t_ (mg L^−1^) is the dye concentration at time t in the solution, V is the solution volume (L) and M is the mass of the biosorbent (g).

The percentage of MB removal was calculated using the following equation:
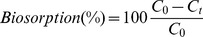
(5)


Different kinetic models such as pseudo-first order (Equation 6), pseudo-second order (Equation 7) and intra-particle diffusion models (Equation 8) were assessed in order to study the rate of MB adsorption over DAB [21], [22], [23].
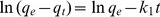
(6)




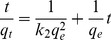
(7)




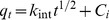
(8)where q_e_ and q_t_ are the amount of MB adsorbed per unit mass (mg g^−1^) at the equilibrium and at time t, respectively while k_1_ (min^−1^)_,_ k_2_ (g mg^−1^ min^−1^) and k_int_ (mg g^−1^ min^−1/2^) are the rate constants whereas C_i_ represents the intercept (mg g^−1^).

### ANN modeling

Artificial neural network (ANN) modeling is important to identify the complex input-output relationship and to develop a model to predict the output of dependent variables from the given set of independent variables [24]. ANN has been used to evaluate the performance of various parameters for dye and metal adsorption using mean square error and regression [25], [26], [27]. The present study uses Neural Network Toolboxof MATLAB R2013a software with a total of 527 experimental data sets and ranges of input variables like pH (2–9), initial dye concentration (25–125 mg L^−1^), adsorbent concentration (1–10 g L^−1^) temperature (293.16–323.16 K), agitation speed (0–150 rpm) and contact time (0–1440 minutes).

The data sets were normalized in the range 0.1–0.9 using the following equation:
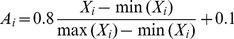
(9)where min(X_i_) and max(X_i_) are the extreme values of variable X_i_ [24].

All data were divided into training (70%), validation (15%), test (15%) subsets and the network was trained with Levenberg-Marquardt back-propagation algorithm. Optimum number of hidden nodes was 10 for the present model. The performance of network was measured by mean squared error (MSE) using following equation:
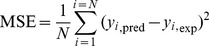
(10)where y_i_,_pred_ and y_i_,_exp_ are the values predicted by the neural network and obtained by experiments, respectively. N is the number of data points and i is an index of data.

Relative importance of different input variables on adsorption efficiency was determined by sensitivity analysis [28] calculated by following formula:
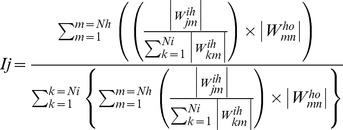
(11)where I_j_ is the relative importance of the j^th^ input variable on the output variable, N_i_ and N_h_ are the numbers of input and hidden neurons, respectively. W is connection weight, the superscripts ‘i’, ‘h’ and ‘o’ refer to input, hidden and output layers, respectively, and the subscripts ‘k’, ‘m’ and ‘n’ refer to input, hidden and output neuron numbers, respectively.

### Column experiment

For the column experiment, a sintered glass disc column (1.2×15 cm) was packed with a known quantity (0.3 g) of DAB biosorbent upto a height of 1 cm. A column feeding solution containing 100 mg L^−1^ MB at pH 7 was loaded into the column at a flow rate of 0.37 ml min^−1^. The fractions were collected at an interval of one hour and concentration of MB was analyzed in each fraction as detailed above. The graph plotted between C/C_0_ and time (t) is termed as the breakthrough curve. Breakthrough (t_b_) and exhaustion (t_e_) times represent the time at which the dye concentration in the collected fractions reached 5% and 95% of feed dye concentration respectively. The length of mass transfer zone (L_m_), mass of dye removed (M_r_) and dye uptake (q_e_) at exhaustion time were calculated using the following formulae [29], [30].
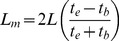
(12)




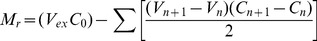
(13)




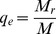
(14)where L (cm) is total bed depth, V_ex_ (L) throughput volume at column exhaustion, C_0_ (mg L^−1^) influent dye concentration, V_n_ (L) throughput volume at n^th^ reading, V_n+1_ throughput volume at (n+1)^th^ reading, C_n_ (mg L^−1^) the effluent dye concentration at n^th^ reading, C_n+1_ (mg L^−1^) the effluent dye concentration at (n+1)^th^ reading and M (g) is the weight of biosorbent used to prepare the column bed.

The length of unused (LUB) and used bed (UB) at breakthrough point were determined by following equations [31].
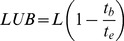
(15)




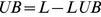
(16)


## Results and Discussion

### Characterization of the biosorbent

**Figure 1:**
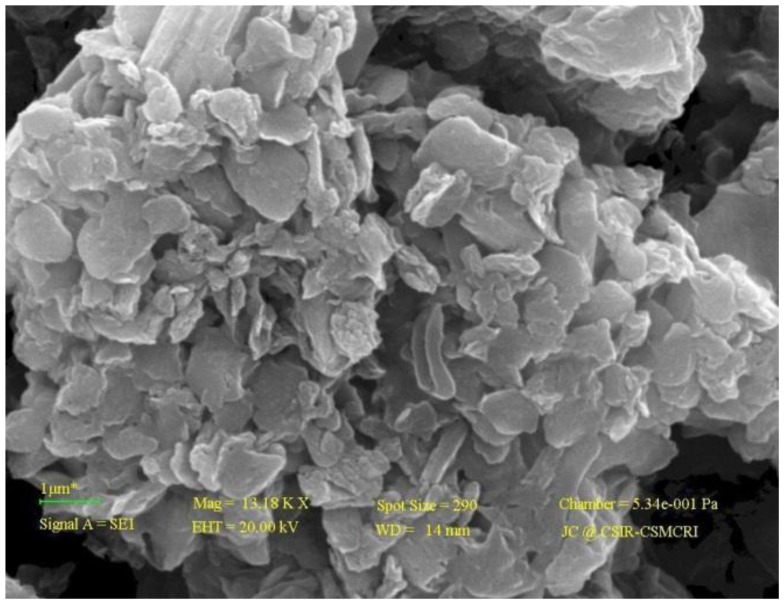
SEM image of DAB.

The texture of the DAB surface was observed by SEM image (Figure 1) which reveals different irregularities leading to surface roughness that plays an important role in the surface adsorption of the dye. Lipid extraction of biomass through solvent leads to harsh effects like breaking of cell wall which may cause such surface topology.

**Figure 2:**
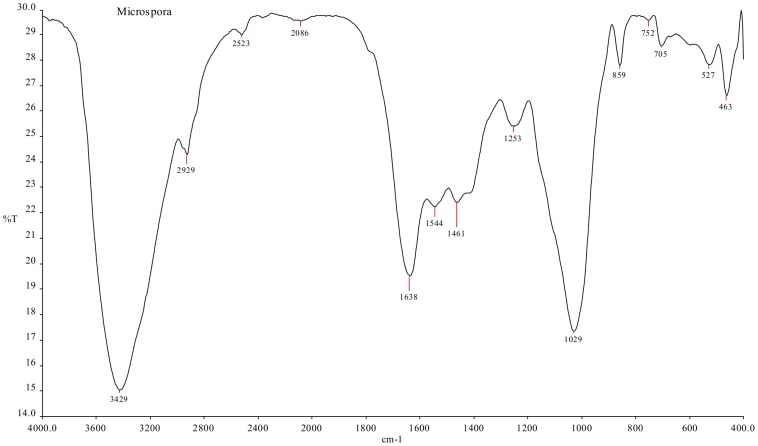
FT-IR spectrum of the DAB.

FT-IR analysis of the DAB (Figure 2) verifies the functional groups present on its surface. Several peaks like 3429 cm^−1^, 2929 cm^−1^, 1461 cm^−1^, 1638 cm^−1^, 1544 cm^−1^ and 1253 cm^−1^ corresponds to the O-H stretching vibrations of glucose, –NH groups of proteins, C–H aliphatic stretching vibrations, C–H scissoring, amide I (carbonyl group stretching), amide II and amide III (NH bending and CN stretching), respectively, confirming the presence of polysaccharides, proteins, etc. [32], [33]. These molecules have their own functional groups such as amino, sulfhydryl, phosphate, carboxylic and thiol groups that can bind various ions [34]. These surface functional groups of biosorbent possibly play an important role in MB sorption.

The particle size distribution of the sample was such that the diameter of 90 and 50% of DAB was less than 1482.57 and 242.75 µm, respectively. The surface area, pore size and pore volume of particles were determined by BET and BJH methods and the values are presented in Table 1. BET and BJH surface area of DAB are 1.46 and 1.57 m^2^ g^−1^ respectively. The BET and BJH pore size of DAB are 11.29 and 24.09 nm respectively, which indicates the porous structure of DAB which aid in MB (size 0.93 nm) sorption [35].

**Table 1:** BET and BJH summary of pore surface area, size and volume.

Area	BET average pore surface area	1.46 m^2^ g^−1^
	BJH adsorption cumulative surface area of pores	1.57 m^2^ g^−1^
	BJH desorption cumulative surface area of pores	1.65 m^2^ g^−1^
Volume	BJH total pore volume	0.004 cm^3^ g^−1^
Pore Size	BET adsorption average pore diameter	11.29 nm
	BJH adsorption average pore diameter	24.09 nm
	BJH desorption average pore diameter	22.46 nm

### Adsorption experiments

#### Effect of pH

**Figure 3:**
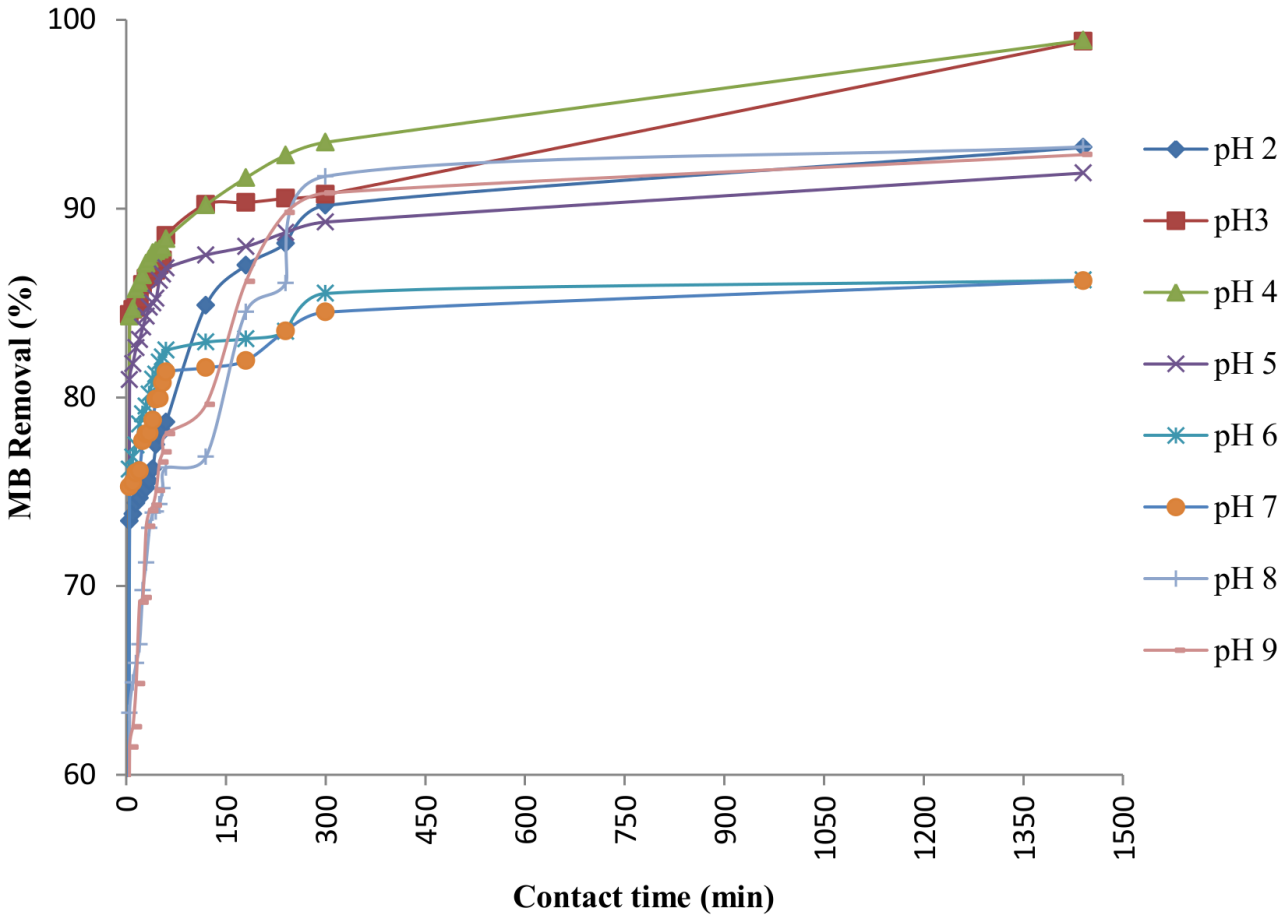
Effect of pH on percentage removal of MB by DAB (10 g L^−1^ DAB, 50 mg L^−1^ MB).

The experiments were carried out at various pH from 2 to 9 (Figure 3). The results were best with approximately 98% MB removal after 24 hours at pH 3 and 4 while MB removal was reduced from 98 to 86% at pH 5–7. MB removal improved from 86 to 93% at pH 7–9. These results prove that MB removal was above 90% for both acidic and basic pH. MB removal was 86% at neutral pH, a condition of natural water bodies, so apparently there is no need to change the pH for further scale up and all further experiments were also conducted at pH 7. Similar trends were observed in MB removal by the alga *Spirogyra* [36], acid activated carbon [37], activated carbon [4], [38].

The point of zero charge (pH_PZC_) is an important parameter for biosorbent to characterize the sensitivity to the pH and their surface charges. The pH_PZC_ of DAB was found to be 9.05. Generally, adsorption of cations is more favoured at pH >pH_PZC_ while for anions at pH <pH_PZC_ [20]. However, the present study emphasizes the point that electrostatic interactions are not the only reason for this adsorption process. When the pH <pH_ZPC_, the repulsion of similar positively charged DAB surface and MB leads to the entry of MB into the interlayer surface of DAB without being adsorbed at the outer surface [39], [40]. In highly alkaline conditions (pH >9), methylene blue forms aggregates due to its zwitterionic structure in aqueous solutions leading to larger conformation like dimeric and trimeric forms which hinders their entry in the porous surface of the biomass [37]. Other possible reasons for such observations include dye-adsorbent interactions due to hydrogen bonding and hydrophobic- hydrophobic interaction mechanisms. Hence, the pore size and surface area, which remain unaffected by pH changes, also play an important role during the process [4], [38].

#### Effect of contact time

**Figure 4:**
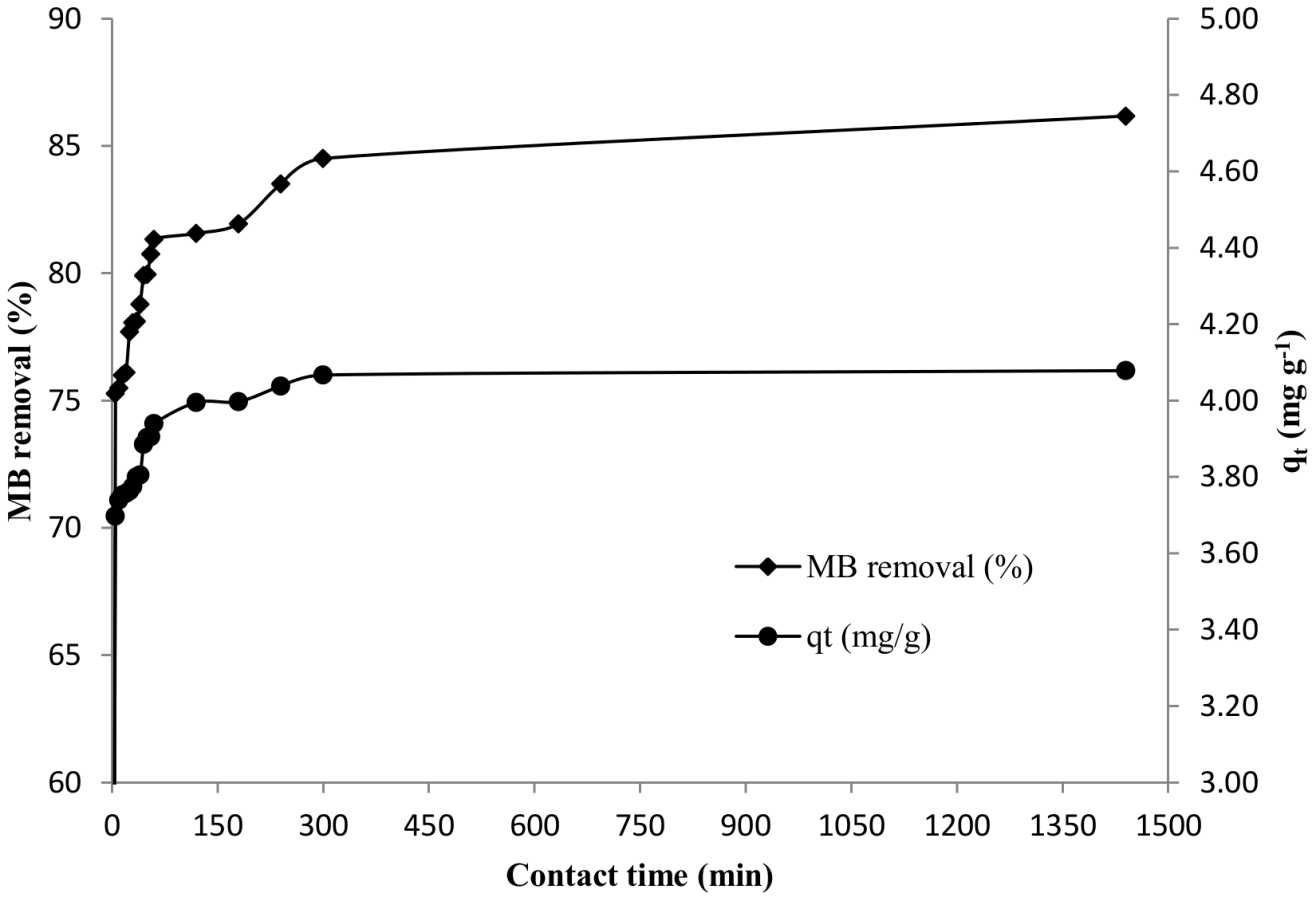
Effect of contact time on percentage removal of MB and adsorption capacity of biosorbent (pH 7, 50 mg L^−1^ MB, 10 g L^−1^ DAB).

Figure 4 represents the effect of contact time on MB removal efficiency and adsorption capacity of DAB. Nearly 75% of MB was removed from the solution by DAB under neutral pH conditions in 5 minutes. The rate of removal was found to decrease gradually with 81 and 86% sorption after an hour and 24 hours respectively. The amount of adsorbed dye (q_e_) increases with an increase in the contact time for up to five hours after which the rate of removal, which is dependent on the number of active sites in the biomass, decreases. A higher concentration of active sites during the initial stages of the process accelerates the removal of the dye from the solution. However, with passage of time the number of active sites get occupied with the dye molecules and hence, the rate of dye removal decreases until attaining equilibrium after 24 hours.

#### Effect of initial methylene blue concentration

**Figure 5:**
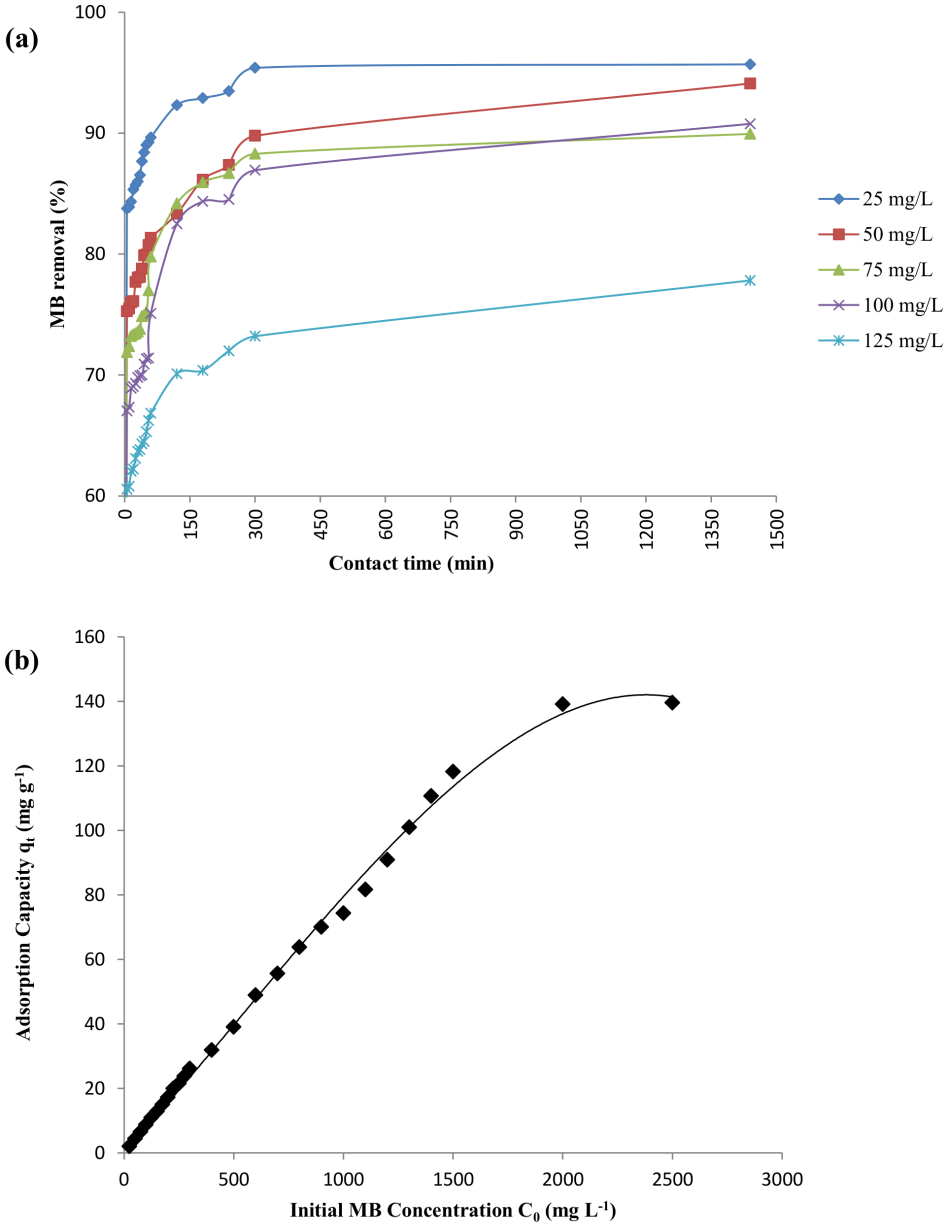
Effect of initial MB concentration on MB biosorption (pH 7, 10 g L^−1^ DAB) (a) Percentage MB removal at different time interval (b) Equilibrium adsorption capacity of DAB at different initial concentrations of MB (10 g L^−1^ DAB).

The effect of initial MB concentration on its removal efficiency by DAB is represented in Figure 5a. The percentage MB removal increases with decrease in initial MB concentration. The equilibrium capacity of DAB linearly increases with increasing MB concentration up to 2000 mg L^−1^ after which the q_e_ value was nearly constant even when the dye concentration was increased upto 2500 mg L^−1^ (Figure 5b). The maximum monolayer adsorption capacity at equilibrium was 139.11 mg g^−1^ at 2000 mg L^−1^. The availability of adsorption sites is the rate-limiting factor which influences the equilibrium concentration of the dye. Higher initial dye concentration in the solution leads to saturation of the available sites much earlier which results in a higher dye content in the solution at equilibrium. Such trends are also observed for algae *Sargassum* sp. [12], [15] and *Galidium* sp. [41] where they have reported a maximum monolayer biosorption capacity of 107.5 and 104 mg g^−1^ respectively.

#### Effect of adsorbent dosage

**Figure 6:**
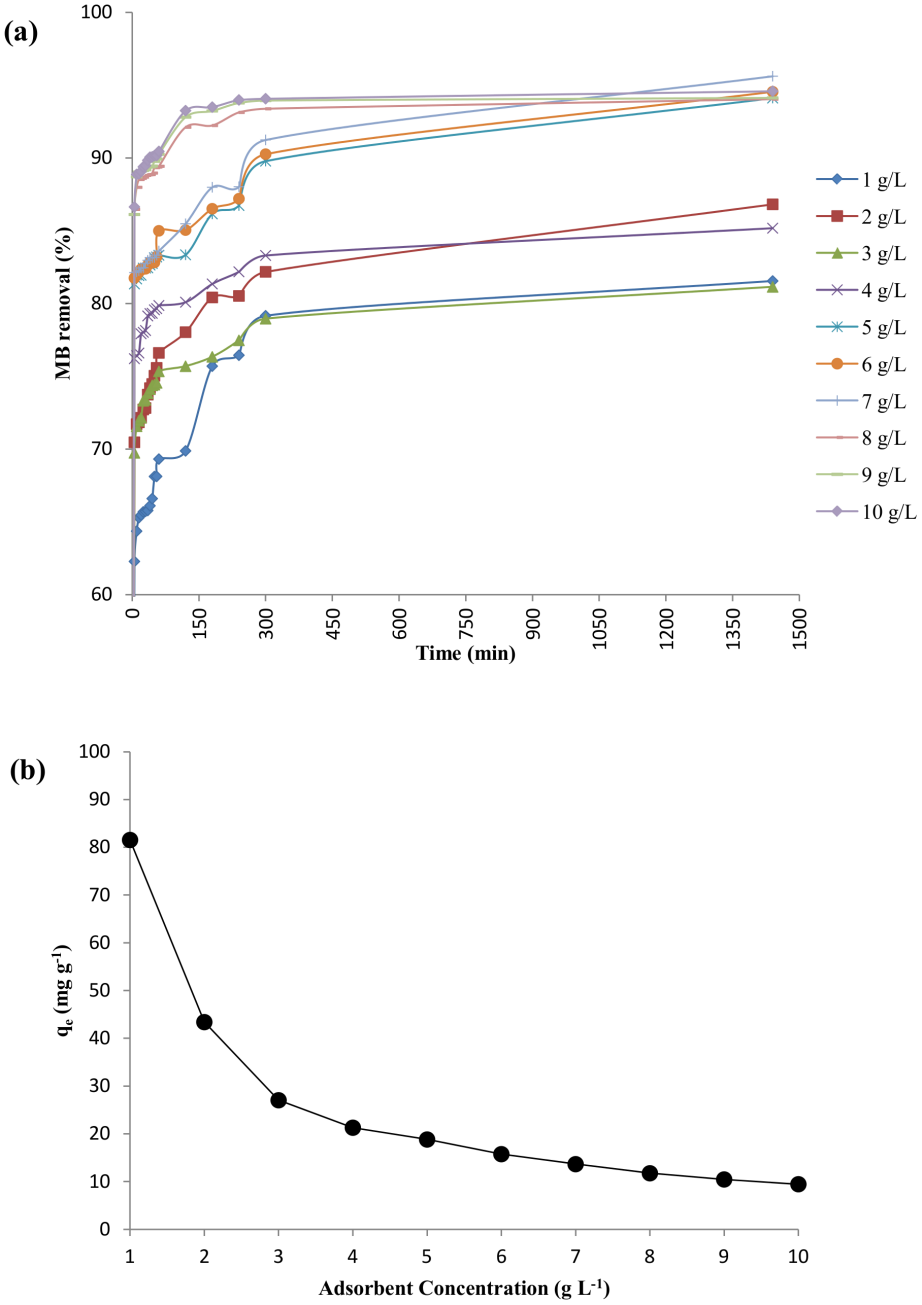
Effect of adsorbent dosage on MB biosorption (pH 7, 100 mg L^−1^ MB). (a) Percentage removal of MB at different time interval (b) Equilibrium adsorption capacity of DAB at different adsorbent concentration.

Figure 6a represents the influence of DAB concentration on the extent of biosorption. The extent of MB removal increased initially with an increase in the DAB concentration. The graph between the dye removal (%) vs. contact time (min) shows that after 24 hours, 94.58% of the dye was removed when we used the adsorbent at a concentration of 10 g L^−1^. The corresponding dye removal values for 5, 6 and 7 g L^−1^ of adsorbent dosage were 94.11, 94.54 and 95.61% respectively. Thus, it is more prudent to use the biosorbent at a concentration of 5–7 g L^−1^ as it results in nearly the same extent of removal at a reduced dosage.

The dye adsorption capacity decreased with increasing biosorbent dosage (Figure 6b). The decreasing value of q_e_ can be attributed to the abundant active sites available for dye sorption when we start increasing the DAB concentration while keeping the concentration of the dye constant. Thus, a low dye: adsorbent ratio results in a lower adsorption capacity. Various other reasons for reduction in q_e_ value have been reported in literature like decrease in the effective surface area of the adsorbent due to partial aggregation of adsorbent particles which forms a protective outer layer preventing the interaction of dye with the biomass and formation of a concentration gradient between the dye solution and the biosorbent [42], [43], [44].

#### Effect of agitation speed

**Figure 7:**
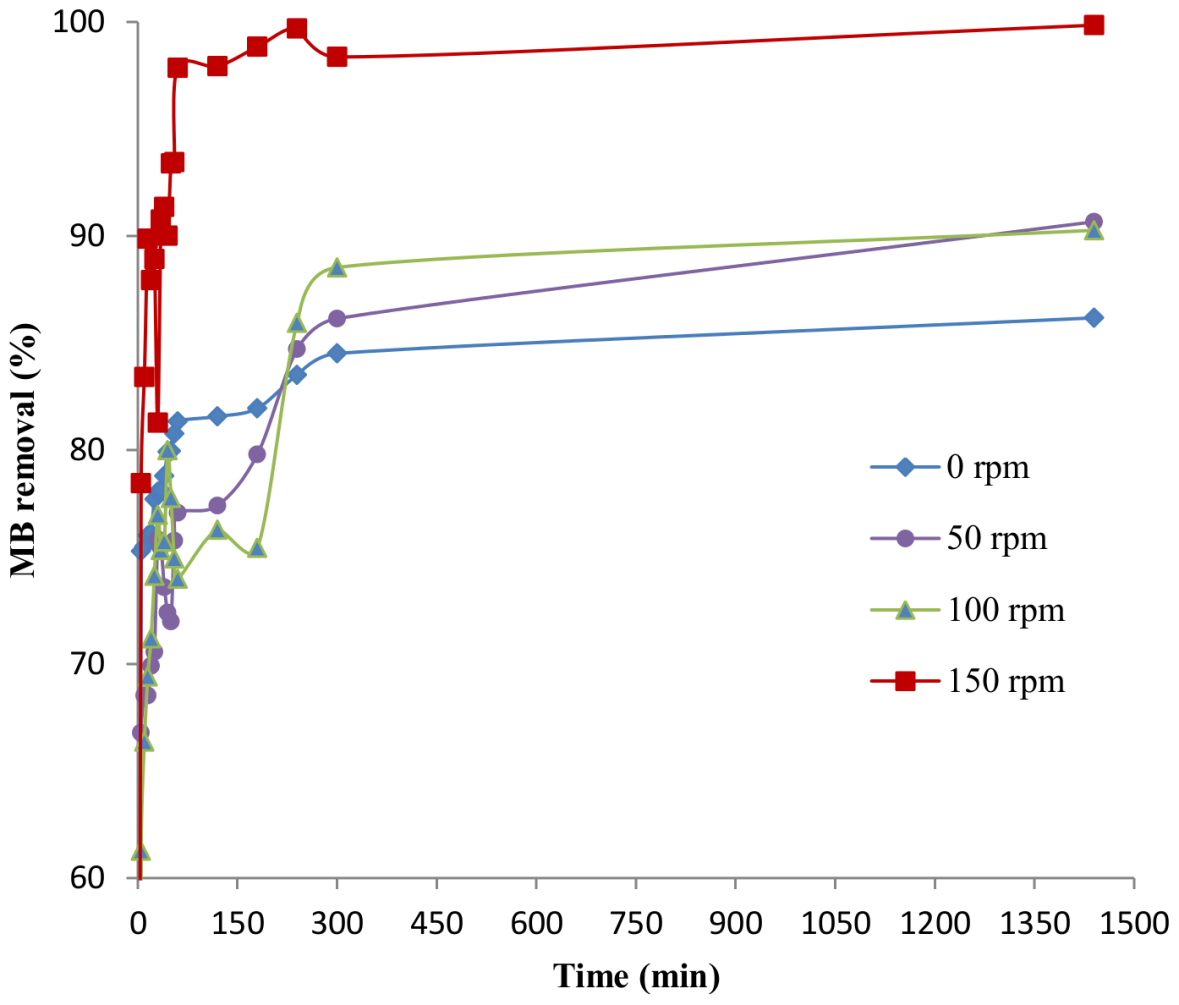
Effect of agitation speed on percentage removal of MB at different time interval (pH 7, 50 mg L^−1^ MB, 10 g L^−1^ DAB).

The distribution of dye molecule in solution is affected by agitation speed and it affects the uptake of dye molecules by disrupting the film resistance surrounding the adsorbent particles [45]. The effect of agitation speed on the rate of MB adsorption is presented in Figure 7. As the speed was increased, the time required to achieve equilibrium was also increased. This phenomenon may be due to the absence of aggregation of the biomass that ultimately increases the available surface area of DAB leading to rapid adsorption of MB. The efficiency of dye removal in 24 hours increases from 86% to 99% as the speed is increased from 0 to 150 rpm. The removal efficiency in 1 hour at 150 rpm is significantly higher than at other speeds supporting the theory of reduced aggregation of biomass at higher speeds.

### Thermodynamic studies

**Figure 8:**
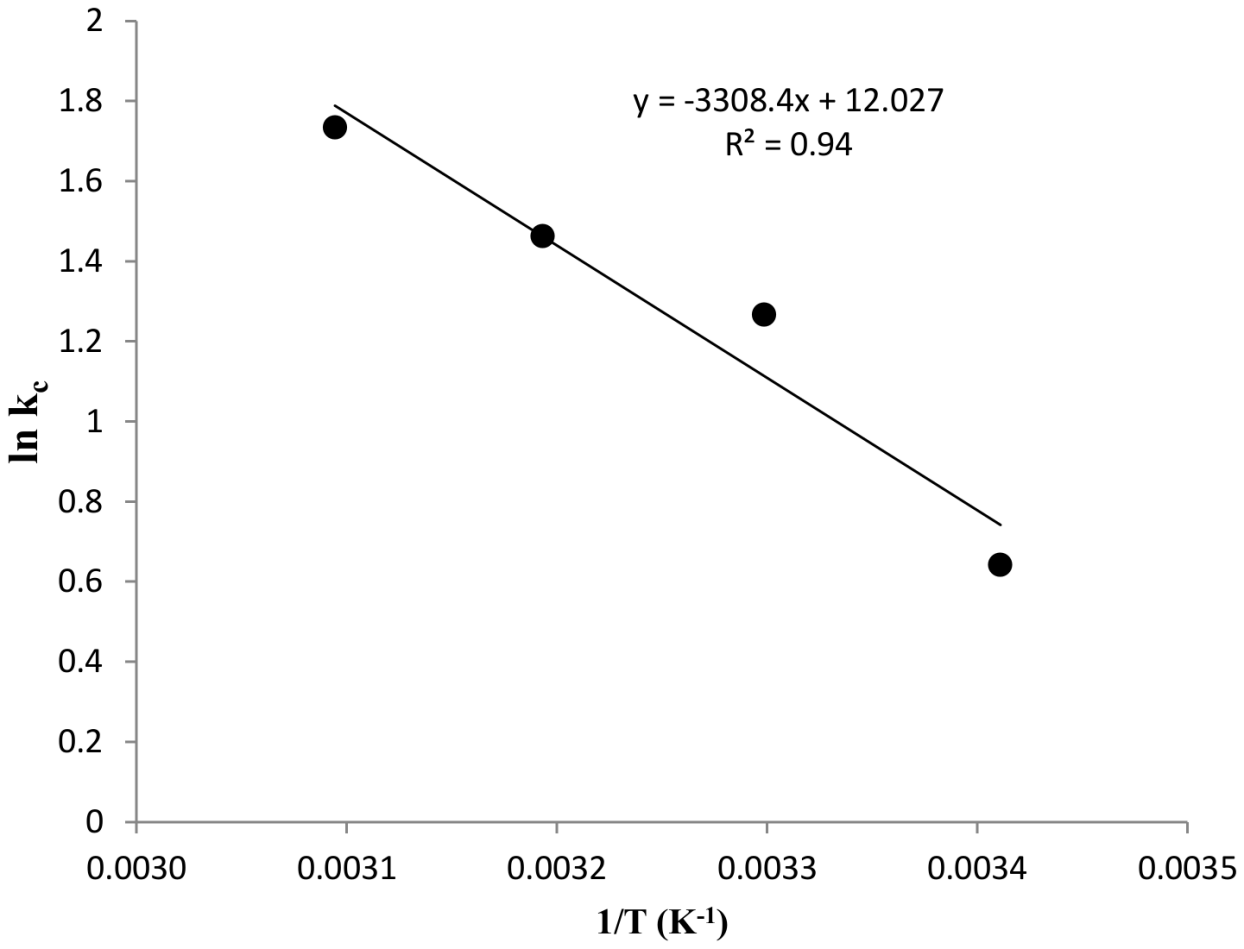
Effect of temperature on MB biosorption illustrated as a van’t Hoff plot (pH 7, 50 mg L^−1^ MB, 10 g L^−1^ DAB, 5 hours contact time).

The effect of temperature on MB adsorption under different temperature regimes (293.16–323.16 K) is depicted in Figure 8. The values for ΔG°, ΔH° and ΔS° are shown in Table 2. As evident from the positive values of ΔH° and ΔS°, the process is spontaneous and endothermic in nature [46]. The negative values of ΔG° also depict the spontaneity of the process. As the temperature is increased (293.16–323.16 K), the values of ΔG° become more negative, indicating that the process becomes more spontaneous. The low value of ΔH° also indicates that physical binding forces may be involved in the process [47]. Increasing the temperature increase the rate of diffusion of dye molecule into the internal pores of the biosorbent [48].

**Table 2:** Thermodynamic parameters of MB biosorption by DMB.

Temperature (K)	ΔG° (kJ mol^−1^)	ΔH° (kJ mol^−1^ K^−1^)	ΔS° (J mol^−1^ K^−1^)
293.16	–1.79	27.50	99.93
303.16	–2.79		
313.16	–3.79		
323.16	–4.79		

### Adsorption kinetics

**Figure 9:**
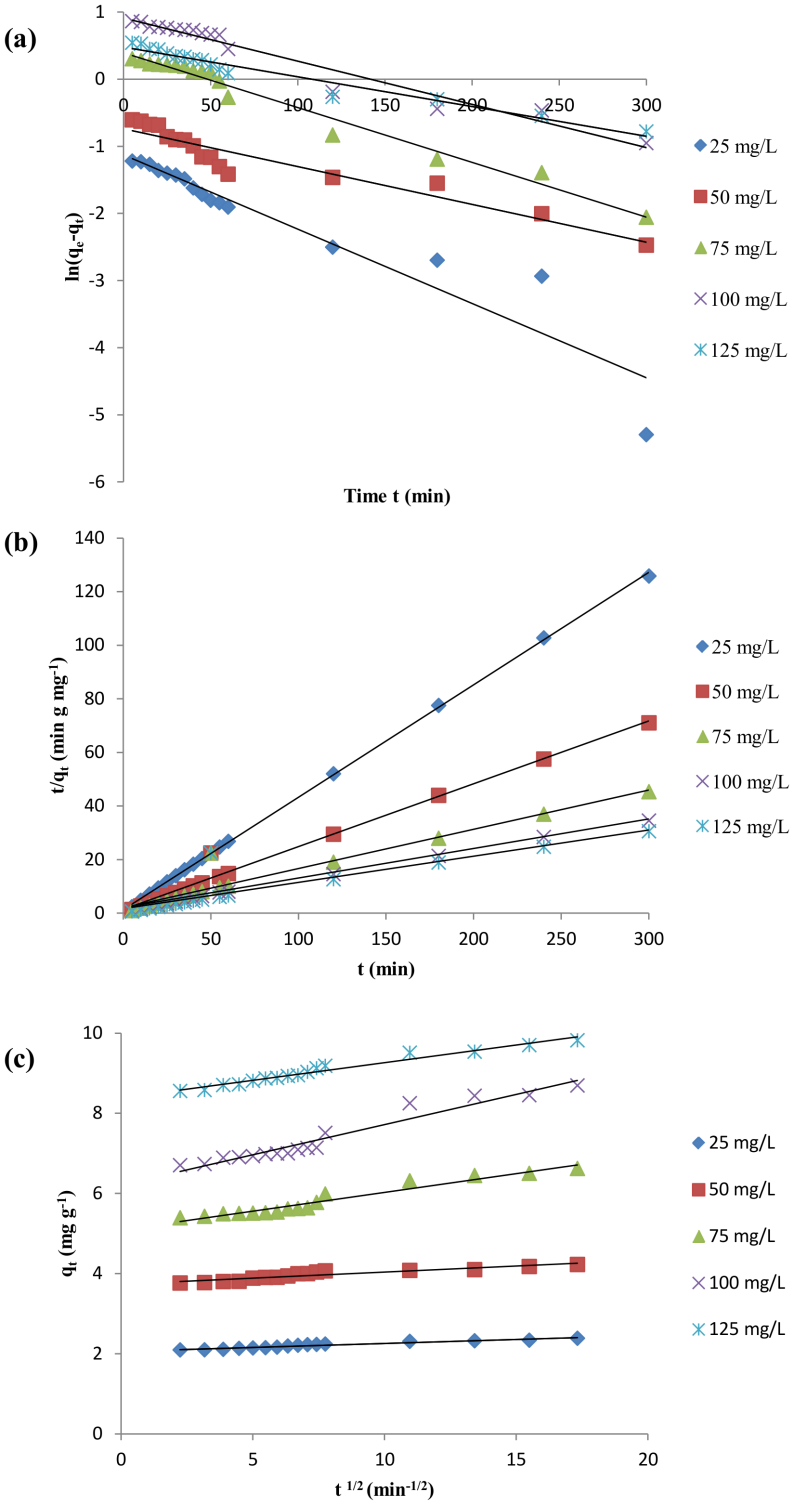
Different kinetic models of MB biosorption. (a) Pseudo-first order (b) Pseudo-second order (c) Intra particle diffusion.

The kinetic data obtained from the effect of initial dye concentration on equilibrium biosorption capacity of biosorbent was utilized for testing different kinetic models. Their graphical presentations are shown in Figure 9. Using the slopes and intercepts, different kinetic parameters i.e. equilibrium biosorption capacity (q_e, cal_) and correlation coefficient (R^2^) values were calculated and are presented in Table 3. Figure 9a shows t vs. ln (q_e_–q_t_) plot for pseudo-first order kinetics. As is evident, the value of the rate constant k_1_ decreases with an increase in initial MB concentration. Most of the literatures cite pseudo second order model as a best fit only at the preliminary stages of adsorption instead of the whole data range [49]. This is evident from the differing experimental and predicted q_e_ values obtained from this model. The t vs. t/q_t_ plot given in Figure 9b represents the pseudo-second order kinetics with the slope equal to 1/q_e_. Decreasing slope of the plot with increasing initial dye concentration indicates increased adsorption capacity at equilibrium (q_e_), whereas the rate constant k_2_ for the process decreases. The pseudo-second order kinetic is more relevant for the present study which is evident by nearly similar values of experimental and calculated values of q_e_ with the high degree of correlation (R^2^ = 0.99). Based on the pseudo second order model, it is assumed that the rate limiting step may be chemical phenomena involving the exchange of electrons between the sorbate and the sorbent [22]. Intra–particle diffusion kinetic model is represented in Figure 9c which depicts a linear relationship between the amounts of dye adsorbed vs. square root of the contact time. The lines do not pass through the origin, denoting the fact that adsorption process is not limited by intra-particle diffusion alone [10]. The adsorptions of MB on DAB occurs by chemisorption via surface exchange reaction until all the active sites are occupied by the dye, which then diffuses into available pores and any channels present in the biomass.

**Table 3:** Kinetic rate constants of different biosorption models.

Kinetic models	Initial dye concentration C_0_ (mg L^−1^)				
	25	50	75	100	125
q_e,exp_ (mg L^−1^)	2.39	4.31	6.75	9.08	10.28
**Pseudo-first order kinetics**					
q_e, cal_ (mg g^−1^)	0.32	0.48	1.48	2.49	1.61
k_1_ (min^−1^)	0.011	0.005	0.008	0.006	0.004
R^2^	0.89	0.90	0.98	0.96	0.96
**Pseudo-second order kinetics**					
q_e,cal_ (mg g^−1^)	2.39	4.27	6.85	9.09	10.31
k_2_ (g mg^−1 ^min^−1^)	0.145	0.041	0.011	0.006	0.006
R^2^	0.99	0.98	0.93	0.85	0.80
**Intra-particle diffusion model**					
C_i, cal_ (mg g^−1^)	2.06	3.73	5.09	6.21	8.38
k_int_ (mg g^−1^ min^−1/2^)	0.02	0.303	0.094	0.15	0.088
R^2^	0.94	0.88	0.94	0.94	0.97

### ANN modeling

**Figure 10:**
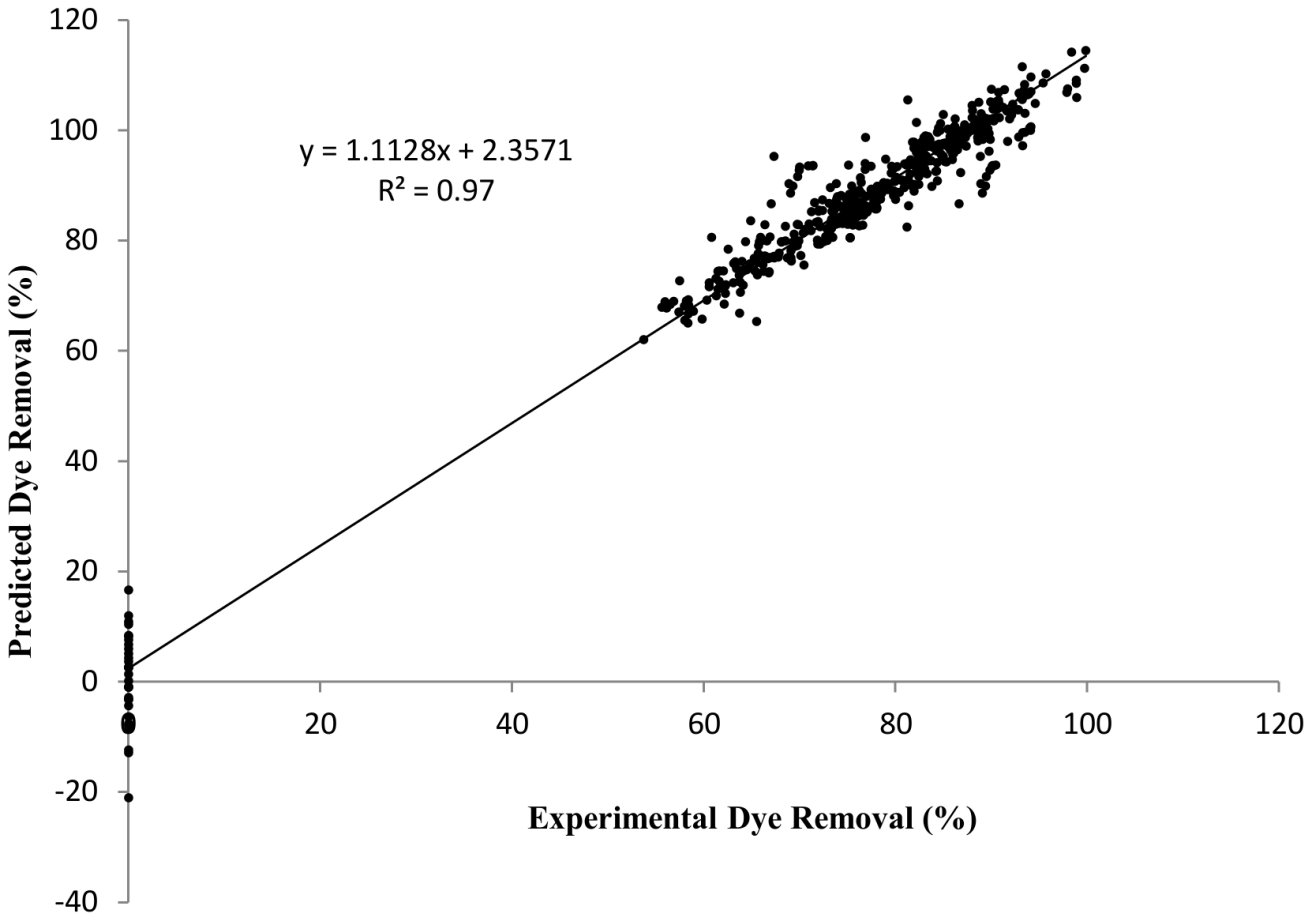
Regression analysis of experimental results and predicted results using artificial neural network.

Figure 10 shows the regression analysis between the experimental and predicted values of adsorption efficiency using a neural network model. The correlation coefficient of this comparison plot was 0.97 which shows that ANN model fits the experimental values of adsorption efficiency (%) and is well reproduced in this system. Similar results were reported for algae *Chara* sp. [27], walnut husk [50] and *Penicilium* sp. [51] with a correlation coefficient ranging from 0.97 to 0.99. The minimum mean squared error for test was 0.0024 at epoch 117.

The relative importance and ranking of the input variables for the dye removal efficiency (%), shown in Table 4, follows the trend:

**Table 4:** Relative importance and ranking of input variables on the dye removal efficiency.

Input variables	Relative Importance (%)	Ranking of inputs as per relative importance
Contact time	18.80	3
pH	15.06	4
Adsorbent Dosage	10.57	6
Initial Dye concentration	10.88	5
Agitation speed	21.08	2
Temperature	23.62	1

Temperature>agitation speed>contact time>pH>Initial dye conc.>Adsorbent dose. The temperature and agitation speed are most influential parameter in biosorption which is supported by literature [52], [53] while contact time and pH have moderate influence on biosorption i.e.18.80% and 15.06% respectively. Such influence was supported by experimental results obtained in sections of respective parameters.

### Column experiments

**Figure 11:**
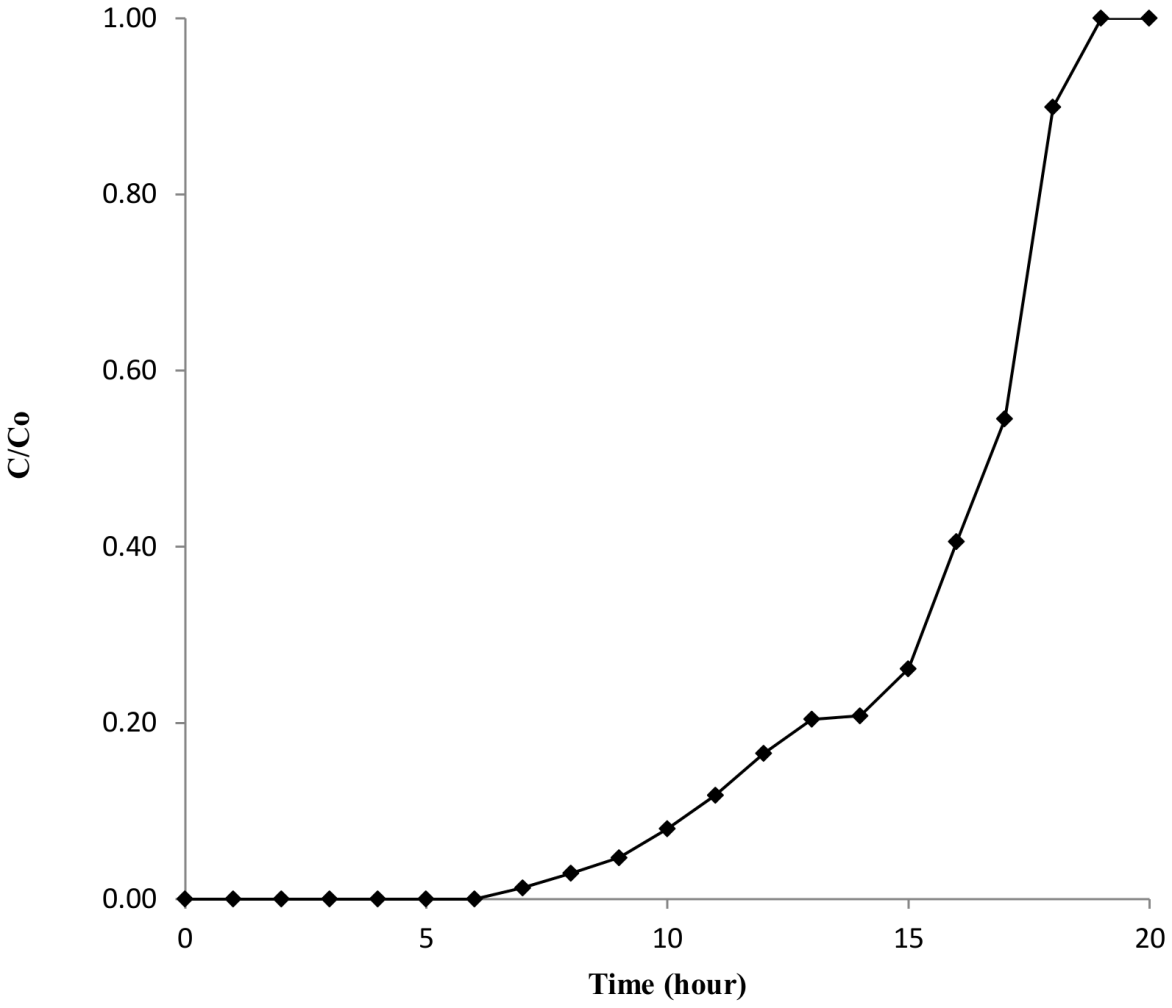
Breakthrough curve of biosorption of MB by DAB.

Adsorption of dye on a fixed bed column is widely used to scale up the process in actual conditions. In the column, when the dye solution enters the bed, it comes in contact with the first few layers of DAB particles and fills up the available active sites. As soon as the adsorbent in the uppermost layer is saturated and the dye solution penetrates further into the bed, the adsorption region shifts down. The bed is considered ineffective when the unabsorbed dye emerges into the effluent. The breakthrough curve of MB adsorption on DAB is represented in Figure 11. The breakthrough time t_b_ and exhaustion time t_e_ were observed to be 7 and 18.5 hours, respectively. The length of mass transfer zone (L_m_), mass of dye removed (M_r_) and dye uptake (q_e_) at exhaustion time were 0.90 cm, 32.27 mg, and 107.57 mg g^−1^ respectively. The length of unused and used beds at breakthrough is 0.62 and 0.38 cm, respectively. In comparison to batch biosorption, column biosorption capacity is much higher because of greater dye concentration gradient at the interface zone of dye solution and biomass bed [54].

## Conclusion

The maximum MB removal was found at both acidic and basic pH but it was also good at neutral pH with 86% dye removal which represents conditions of natural water bodies. The DAB (10 g L^−1^) removes 86% of the dye (50 mg L^−1^) in 5 minutes under static condition and nearly 100% in 24-hours with agitation at 150 rpm, which indicates the MB removal by DAB in static conditions is more feasible as it requires lesser energy input. The optimum DAB concentration was 5–7 g L^−1^ for effective MB removal at 100 mg L^−1^ MB concentration. The highest adsorption capacity value of DAB is 139.11 mg g^−1^ at 2000 mg L^−1^ initial MB concentration while 107.57 mg g^−1^ for column study at 100 mg L^−1^ MB concentration in preliminary study. The kinetic studies of MB adsorption were best fit with the pseudo-second order model. Based on the study of different parameters and kinetic models it can be concluded that no single rate limiting step is involved in biosorption mechanism. It involved different bonding of surface active sites functional group with dye, physical adsorption of dye in micro-pores and macro-pores of DAB surface and monolayer adsorption. ANN modelling for each parameter predicts the experimental value with high correlation coefficient value (R^2^ = 0.97) and sensitivity analysis suggests that temperature and agitation speed are the most influencing parameter in this biosorption experiment. Present study supports the practical valorisation of de-oiled algal biomass, obtained during algal biofuel process, for the treatment of coloured effluents and economically balances the algal bio-refinery. The advantages of the present work include the utilization of DAB as a biosorbent for removal of hazardous dyes, utilizing the waste generated from algal biofuel as well as reducing algal blooms.

## References

[1] Chen KC, Wu JY, Liou DJ, Hwang SCJ (2003) Decolorization of the textile dyes by newly isolated bacterial strains. J Biotechnol 101: 57–68. 12523970 10.1016/s0168-1656(02)00303-6

[2] Gong R, Ding Y, Li M, Yang C, Liu H, et al (2005) Utilization of powdered peanut hull as biosorbent for removal of anionic dyes from aqueous solution. Dyes Pigments 64: 187–192. 15707341

[3] Vadivelan V, Kumar KV (2005) Equilibrium, kinetics, mechanism, and process design for the sorption of methylene blue onto rice husk. J Colloid Interf Sci 286: 90–100. 10.1016/j.jcis.2005.01.007 15848406

[4] Karaca S, Gürses A, Açıkyıldız M, Ejder (Korucu) M (2008) Adsorption of cationic dye from aqueous solutions by activated carbon. Micropor Mesopor Mat 115: 376–382.

[5] Kannan N, Sundaram MM (2001) Kinetics and mechanism of removal of methylene blue by adsorption on various carbons - a comparative study. Dyes Pigments 51: 25–40.

[6] Sarma GK, SenGupta S, Bhattacharyya KG (2011) Methylene blue adsorption on natural and modified clays. Separ Sci Technol 46: 1602–1614.

[7] Hao OJ, Kim H, Chiang PC (2000) Decolorization of wastewater. Crit Rev Env Sci Tec 30: 449–505.

[8] Low KS, Lee CK, Tan KK (1995) Biosorption of basic dyes by water hyacinth roots. Bioresource Technol 52: 79–83.

[9] Lee CK, Low KS, Chung LC (1997) Removal of some organic dyes by hexane-extracted spent bleaching earth. J Chem Technol Biotechnol 69: 93–99.

[10] Banat F, Al-Asheh S, Al-Makhadmeh L (2003) Evaluation of the use of raw and activated date pits as potential adsorbents for dye containing waters. Process Biochem 39: 193–202.

[11] Rahman IA, Saad B (2003) Utilization of guava seeds as a source of activated carbon for removal of methylene blue from aqueous solution. Malays J Chem 5: 8–14.

[12] Rubin E, Rodriguez P, Herrero R, Cremades J, Barbara I, et al (2005) Removal of methylene blue from aqueous solutions using as biosorbent *Sargassum muticum*: an invasive macroalga in Europe. J Chem Technol Biotechnol 80: 291–298.

[13] Rajeshwarisivaraj, Subburam V (2002) Activated parthenium carbon as an adsorbent for the removal of dyes and heavy metal ions from aqueous solution. Bioresource Technol 85: 205–206. 10.1016/s0960-8524(02)00089-5 12227547

[14] Fu Y, Viraraghavan T (2001) Fungal decolorization of dye wastewaters: a review. Bioresource Technol 79: 251–262. 10.1016/s0960-8524(01)00028-1 11499579

[15] Kumar PS, Pavithra J, Suriya S, Ramesh M, Kumar KA (2014) *Sargassum wightii*, a marine alga is the source for the production of algal oil, bio-oil, and application in the dye wastewater treatment. Desalin Water Treat: 1–17.

[16] Chisti Y (2007) Biodiesel from microalgae. Biotechnol Adv 25: 294–306. 17350212 10.1016/j.biotechadv.2007.02.001

[17] Darzins A, Pienkos P, Edye L (2010.) Current status and potential for algal biofuels production. A report to IEA Bioenergy Task, 39. National Renewable Energy Laboratory, Golden, Colorado.

[18] Rashid N, Rehman MSU, Han JI (2013) Recycling and reuse of spent microalgal biomass for sustainable biofuels. Biochem Eng J 75: 101–107.

[19] Mishra S, Ghosh PK, Gandhi M, Bhattacharyya S, Maiti S, et al. (2014) Engine worthy fatty acid methyl ester (biodiesel) from naturally occurring marine microalgal mats and marine microalgae cultured in open salt pans together with value addition of co-products. Patent Application US20140099684 A1.

[20] Sadaf S, Bhatti HN, Ali S, Rehman K (2013) Removal of Indosol Turquoise FBL dye from aqueous solution by bagasse, a low cost agricultural waste: batch and column study. Desalin Water Treat 52: 184–198.

[21] Lagergren S (1898) About the theory of so-called adsorption of soluble substances. Kungliga Svenska Vetenskapsakademiens Handlingar 24: 1–39.

[22] Ho YS, McKay G (1999) Pseudo-second order model for sorption processes. Process Biochem 34: 451–465.

[23] Weber WJ, Morris JC (1963) Kinetics of adsorption on carbon from solution. J Sanit Eng Div Proc Am Soc Civ Eng 89: 31–60.

[24] Ibrahim OM (2013) A comparison of methods for assessing the relative importance of input variables in artificial neural networks. J Appl Sci Res 9: 5692–5700.

[25] Aleboyeh A, Kasiri MB, Olya ME, Aleboyeh H (2008) Prediction of azo dye decolorization by UV/H_2_O_2_ using artificial neural networks. Dyes Pigments 77: 288–294.

[26] Aber S, Amani-Ghadim AR, Mirzajani V (2009) Removal of Cr(VI) from polluted solutions by electrocoagulation: Modeling of experimental results using artificial neural network. J Hazard Mater 171: 484–490. 19589640 10.1016/j.jhazmat.2009.06.025

[27] Khataee AR, Dehghan G, Ebadi A, Zarei M, Pourhassan M (2010) Biological treatment of a dye solution by Macroalgae *Chara* sp.: Effect of operational parameters, intermediates identification and artificial neural network modeling. Bioresource Technol 101: 2252–2258. 10.1016/j.biortech.2009.11.079 20022487

[28] Garson G (1991) Interpreting neural-network connection weights. Artif Intell Expert 6: 46–51.

[29] Fernandez ME, Nunell GV, Bonelli PR, Cukierman AL (2012) Batch and dynamic biosorption of basic dyes from binary solutions by alkaline-treated cypress cone chips. Bioresource Technol 106: 55–62. 10.1016/j.biortech.2011.12.003 22197337

[30] Pota AA, Mathews AP (2000) Adsorption dynamics in a stratified convergent tapered bed. Chem Eng Sci 55: 1399–1409.

[31] Otero M, Zabkova M, Rodrigues AE (2005) Comparative study of the adsorption of phenol and salicylic acid from aqueous solution onto nonionic polymeric resins. Sep Purif Technol 45: 86–95.

[32] Ashkenazy R, Gottlieb L, Yannai S (1997) Characterization of acetone-washed yeast biomass functional groups involved in lead biosorption. Biotechnol Bioeng 55: 1–10. 18636438 10.1002/(SICI)1097-0290(19970705)55:1<1::AID-BIT1>3.0.CO;2-H

[33] Sindaraganesan N, Ilakiamani S, Saleem H, Mohan S (2004) FT-Raman, FTIR spectra and normal coordinate analysis of 5-bromo-2-nitropyridine. Indian J Pure Ap Phy 42: 585–590.

[34] Dheetcha A, Mishra S (2008) Biosequestering potential of *Spirulina platensis* for uranium. Curr Microbiol 57: 508–514. 18825456 10.1007/s00284-008-9277-7

[35] Tanada S, Kita T, Boki K, Tamura T, Murai Y (1980) Mechanism of adsorption of methylene blue on magnesium silicate. Chem Pharm Bull 28: 2503–2506.

[36] Mohan SV, Ramanaiah SV, Sarma PN (2008) Biosorption of direct azo dye from aqueous phase onto Spirogyra sp. I02: Evaluation of kinetics and mechanistic aspects. Biochem Eng J 38: 61–69.

[37] Arivoli S, Hema M, Parthasarathy S, Manju N (2010) Adsorption dynamics of methylene blue by acid activated carbon. J Chem Pharm Res 2: 626–641.

[38] Al-Degs YS, El-Barghouthi MI, El-Sheikh AH, Walker GM (2008) Effect of solution pH, ionic strength, and temperature on adsorption behavior of reactive dyes on activated carbon. Dyes Pigments 77: 16–23.

[39] Hisarli G (2005) The effects of acid and alkali modification on the adsorption performance of fuller’s earth for basic dye. J Colloid Interf Sci 281: 18–26. 10.1016/j.jcis.2004.08.089 15567375

[40] Guo Y, Zhao J, Zhang H, Yang S, Qi J, et al (2005) Use of rice husk-based porous carbon for adsorption of Rhodamine B from aqueous solutions. Dyes Pigments 66: 123–128.

[41] Vilar VJP, Botelho CMS, Boaventura RAR (2007) Methylene blue adsorption by algal biomass based materials: Biosorbents characterization and process behaviour. J Hazard Mater 147: 120–132. 17240055 10.1016/j.jhazmat.2006.12.055

[42] Ponnusami V, Vikram S, Srivastava SN (2008) Guava (*Psidium guajava*) leaf powder: Novel adsorbent for removal of methylene blue from aqueous solutions. J Hazard Mater 152: 276–286. 17692457 10.1016/j.jhazmat.2007.06.107

[43] Garg VK, Amita M, Kumar R, Gupta R (2004) Basic dye (methylene blue) removal from simulated wastewater by adsorption using Indian Rosewood sawdust: a timber industry waste. Dyes Pigments 63: 243–250.

[44] Kumar KV, Porkodi K (2007) Mass transfer, kinetics and equilibrium studies for the biosorption of methylene blue using *Paspalum notatum* . J Hazard Mater 146: 214–226. 17222969 10.1016/j.jhazmat.2006.12.010

[45] Al-Ghouti MA, Khraisheh MAM, Ahmad MNM, Allen S (2009) Adsorption behaviour of methylene blue onto Jordanian diatomite: A kinetic study. J Hazard Mater 165: 589–598. 19022576 10.1016/j.jhazmat.2008.10.018

[46] Sharma YC, Uma, Upadhyay SN (2009) Removal of a cationic dye from wastewaters by adsorption on activated carbon developed from coconut coir. Energy Fuels 23: 2983–2988.

[47] Gunasekar V, Ponnusami V (2012) Kinetics, equilibrium, and thermodynamic studies on adsorption of methylene blue by carbonized plant leaf powder. J Chem 2013.

[48] Khodaie M, Ghasemi N, Moradi B, Rahimi M (2013) Removal of methylene blue from wastewater by adsorption onto ZnCl_2_ activated corn husk carbon equilibrium studies. J Chem 2013.

[49] Sadaf S, Bhatti HN (2014) Batch and fixed bed column studies for the removal of Indosol Yellow BG dye by peanut husk. J Taiwan Inst Chem Eng 45: 541–553.

[50] Çelekli A, Birecikligil SS, Geyik F, Bozkurt H (2012) Prediction of removal efficiency of Lanaset Red G on walnut husk using artificial neural network model. Bioresource Technol 103: 64–70. 10.1016/j.biortech.2011.09.106 22018750

[51] Yang Y, Wang G, Wang B, Li Z, Jia X, et al (2011) Biosorption of Acid Black 172 and Congo Red from aqueous solution by nonviable *Penicillium* YW 01: Kinetic study, equilibrium isotherm and artificial neural network modeling. Bioresource Technol 102: 828–834. 10.1016/j.biortech.2010.08.125 20869234

[52] Yang Y, Lin X, Wei B, Zhao Y, Wang J (2014) Evaluation of adsorption potential of bamboo biochar for metal-complex dye: equilibrium, kinetics and artificial neural network modeling. Int J Environ Sci Technol 11: 1093–1100.

[53] Demir G, Dural M, Alyuruk H, Cavas L (2012) Artificial neural network model for biosorption of methylene blue by dead leaves of *Posidonia oceanica* (L.) Delile. Neural Netw World 22: 479–494.

[54] Gupta VK, Mittal A, Krishnan L, Gajbe V (2004) Adsorption kinetics and column operations for the removal and recovery of malachite green from wastewater using bottom ash. Sep Purif Technol 40: 87–96.

